# Osteoporosis is associated with elevated baseline cerebrospinal fluid biomarkers and accelerated brain structural atrophy among older people

**DOI:** 10.3389/fnagi.2022.958050

**Published:** 2022-09-16

**Authors:** Hao Pan, Jiali Cao, Congcong Wu, Furong Huang, Peng Wu, Junzhe Lang, Yangbo Liu

**Affiliations:** ^1^Department of Orthopedics, The First Affiliated Hospital of Wenzhou Medical University, Wenzhou, China; ^2^Department of Outpatient, The First Affiliated Hospital of Wenzhou Medical University, Wenzhou, China

**Keywords:** Alzheimer’s disease, cerebrospinal fluid, biomarker, entorhinal cortex, hippocampus, osteoporosis, cross-sectional study, longitudinal study

## Abstract

**Objective:**

The aim of this study was to examine whether osteoporosis (OP) is associated with Alzheimer’s disease-related cerebrospinal fluid (CSF) biomarkers and brain structures among older people.

**Methods:**

From the Alzheimer’s disease Neuroimaging Initiative database, we grouped participants according to the OP status (OP+/OP−) and compared the Alzheimer’s disease (AD)-related CSF biomarker levels and the regional brain structural volumes between the two groups using multivariable models. These models were adjusted for covariates including age, education, gender, diagnosis of Alzheimer’s disease, and apolipoprotein E4 carrier status.

**Results:**

In the cross-sectional analyses at baseline, OP was related to higher CSF t-tau (total tau) and p-tau_181_ (tau phosphorylated at threonine-181) but not to CSF amyloid-beta (1–42) or the volumes of entorhinal cortex and hippocampus. In the longitudinal analyses, OP was not associated with the change in the three CSF biomarkers over time but was linked to a faster decline in the size of the entorhinal cortex and hippocampus.

**Conclusion:**

OP was associated with elevated levels of CSF t-tau and p-tau_181_ at baseline, and accelerated entorhinal cortex and hippocampal atrophies over time among older people.

## Introduction

An increase in life expectancy causes the aging of the population. The growing older population is plagued by age-related diseases, typically Alzheimer’s disease (AD) and osteoporosis (OP). AD is a well-known neurodegenerative disease that causes cognitive dysfunction among older adults (Mangialasche et al., 2010). OP, on the contrary, is a skeletal disease characterized by low bone mineral density (BMD) and micro-architectural bone tissue deterioration (Black and Rosen, 2016). Although AD and OP appear to have nothing in common, we are not the first to suspect that they may have connections (Yaffe et al., 1999; Tan et al., 2005; Zhou et al., 2014; Sohrabi et al., 2015).

The potential connections have been implicated from a variety of perspectives. Experimental evidence supported that AD was linked to pathogenic changes in osteoporotic animal models (Li et al., 2013; Calabrese et al., 2016), while low BMD was a trait of certain AD mouse models (Xia et al., 2013; Dengler-Crish et al., 2017). According to epidemiological studies, low BMD increases the risk of AD, while OP affects nearly half of cognitively impaired patients (Ebrahimpur et al., 2020). Large-scale investigations focused on the relationship between the two chronic degenerative diseases were first reported by Yaffe et al. (1999) who found that women with osteoporosis have poorer cognitive functions and greater risks of cognitive deterioration. In Germany and South Korea, this relationship was further confirmed for both sexes (Kostev et al., 2018; Kwon et al., 2021). These studies indicated that AD and OP may share some central mechanisms. However, none of them have investigated the long-term pathological connections between OP and AD in living humans.

Pathological evidence has been widely valued in the diagnosis of AD. AD-related cerebrospinal fluid (CSF) biomarkers, including amyloid-beta (Aβ) protein, total tau (t-tau), and phosphorylated tau (p-tau), have been established as core indicators to define the progressive stage in the AD continuum (McKhann et al., 2011; Jack et al., 2016). The aberrant buildup of these pathological proteins will cause generalized atrophy of brain structures, typically affecting the entorhinal cortex and hippocampus (Callen et al., 2001; Killiany et al., 2002; Kril et al., 2002; Dengler-Crish et al., 2017). The degeneration of these brain structures, which are measurable on MRI, causes the progression of AD (Killiany et al., 2002).

Using the Alzheimer’s disease neuroimaging initiative (ADNI) database, this study aims to find out whether OP relates to AD-related CSF biomarkers and brain structures among older people.

## Materials and methods

### Alzheimer’s disease neuroimaging initiative

Raw data were pulled from the ADNI database.^1^ ADNI researchers collect, validate, and utilize data, including demographic characteristics, medical history, images, genetics, cognitive tests, CSF, and blood biomarkers, from participants who are adults aged 55–90 years with AD patients, mild cognitive impairment (MCI) subjects, and elderly normal controls (NC). The ADNI study was approved by the institutional review board at each ADNI center, and informed written consent was obtained from each participant. Further information is available at www.adni-info.org and in previous reports (Jack et al., 2010; Jagust et al., 2010; Petersen et al., 2010; Saykin et al., 2010; Weiner et al., 2010).

### Participants

**FIGURE 1:**
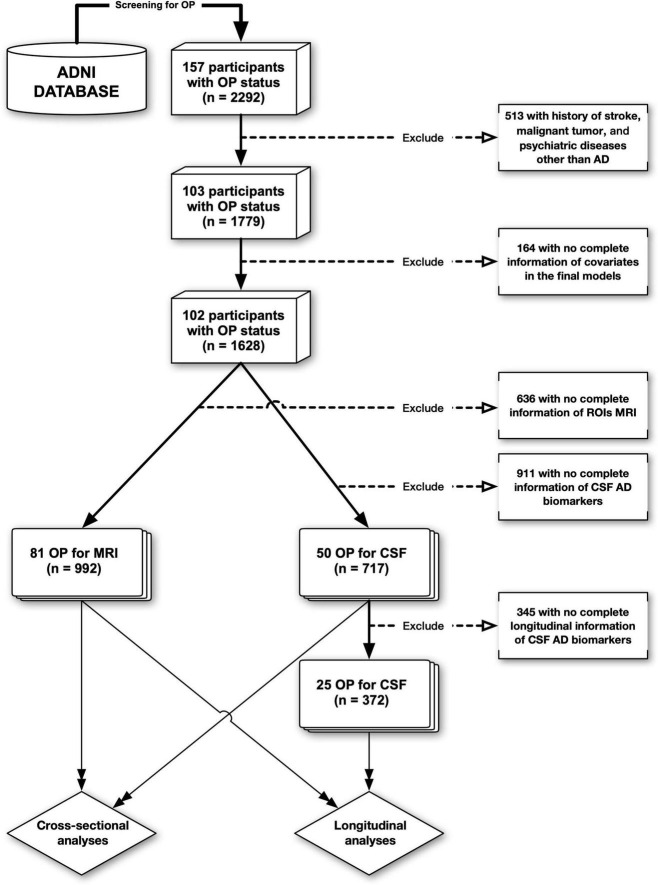
The flowchart of data processing. ADNI, Alzheimer’s disease neuroimaging initiative; OP, osteoporosis; AD, Alzheimer’s disease; CSF, cerebrospinal fluid.

Alzheimer’s Disease Neuroimaging Initiative provides a systematic record of history for each participant (“RECMHIST.csv”), allowing researchers to extract terms using screening techniques. Our screening terms for OP included “OP” or “osteoporo (sis/tic),” which we used to group participants (OP+/OP−). Based on the information in ADNI data, 157 out of 2,292 participants were found to have OP at baseline, among whom, 102 out of 1,628 had complete covariate information and no history of depression, anxiety, malignant tumor, or stroke. Then, the data flow was split into two branches. One was CSF data (*n* = 717), which excluded participants with incomplete Aβ_1–42_, t-tau, and p-tau_181_ (tau phosphorylated at threonine-181) information. The other was MRI data (*n* = 992), which eliminated individuals with incomplete information about intracranial volume (ICV), entorhinal cortex volume (ECV), and hippocampal volume (HV). Besides, to conform the data to the demands of longitudinal analysis—that is, individuals must have at least one valid follow-up record in addition to the baseline one—345 participants were removed from the CSF data for the longitudinal analyses, whereas none were excluded from the MRI data. Figure 1 is the flowchart of the data processing.

### Cerebrospinal fluid measurements

Cerebrospinal fluid Aβ_1–42_, t-tau, and p-tau_181_ were measured using the INNOBIA AlzBio3 immunoassay (Fujirebio, Belgium, pg/ml). To keep the within-batch precision values under 10% (5.1–7.8% for Aβ_1–42_, 4.4–9.8% for t-tau, and 5.1–8.8% for p-tau_181_), ADNI provided unified CSF collection and procedural protocols (Shaw et al., 2009). From the ADNI file (“ADNIMERGE.csv”), we set the measurements of these CSF biomarkers as response variables in our models of CSF data.

### Magnetic resonance imaging measurements

Alzheimer’s disease neuroimaging initiative provides a set of standardized processes for MRI acquisition to reduce systematic error (Christensen et al., 1997; Hsu et al., 2002). The ECV, HV, and ICV were also extracted from the ADNI file (“ADNIMERGE.csv”). Both ECV and HV were normalized by ICV using a residual method to remove head size differences (Voevodskaya et al., 2014; Pintzka et al., 2015). In our models of MRI data, the response variables were ICV normalized ECV and HV (ECV_*n*_ and HV_*n*_, mm^3^).

### Statistical analyses

Demographic, cross-sectional, and longitudinal analyses were conducted on CSF and MRI data. Baseline demographic variables were compared between the OP− and OP+ groups (Chi-square tests for categorical variables; Student’s *t*-tests for normally distributed variables; and Mann–Whitney *U*-tests for non-normally distributed variables). The results of descriptive statistics are presented for the normally distributed variables as “mean [SD]” and for the non-normally distributed variables as “median [P25, P75]” (P25: 25th percentile; P75: 75th percentile).

In the cross-sectional analyses at baseline, we first investigated the associations between OP and CSF biomarkers (Aβ_1–42_, t-tau, and p-tau_181_) after adjusting for covariates including age, sex, education, apolipoprotein E4 (APOE4) carrier status, and diagnosis of AD. Next, we tested the differences in brain structures (ECV_*n*_ and HV_*n*_) between the OP− and OP+ groups *via* linear regression models adjusted for identical covariates.

In the longitudinal analyses, we fitted linear mixed-effects models to characterize individual paths of change. These models had random intercepts and slopes for time and an unstructured covariance matrix for the random effects and included the interaction between (continuous) time and OP status as the predictor. These models were adjusted for the same covariates, including age, sex, education, APOE4 carrier status, and diagnosis of AD.

*P*-values <0.05 were considered to reject the null hypothesis. *P*-values in the linear regression models and linear mixed-effects models were calculated using the Satterthwaite’s degrees of freedom method (“lmerTest” R package), and the Holm’s method (also called the Holm–Bonferroni method) is used to counteract the problem of multiple comparisons. R software (version 4.2.0) and GraphPad Prism (version 9.3.1) were used for statistical analysis and visualization.

## Results

### Demographic variables at baseline

#### Cerebrospinal fluid biomarkers

In the CSF data, at baseline, the measurements of Aβ_1–42_, t-tau, and p-tau_181_ were available for 717 participants, of whom 50 had OP (154 [23.1%] NC, 383 [57.4%] MCI, and 130 [19.5%] AD in OP− group; 8 [16.0%] NC, 28 [56.0%] MCI, and 14 [28.0%] AD in OP + group; *P* = 0.256). Participants without OP were about 1 year younger than those with OP (73.9 [7.2] for OP−, 74.9 [7.0] for OP+; *P* = 0.382). Participants with OP were more likely to be female (42 [84.0%]) than those without OP (female 233 [34.9%]; *P* < 0.001). A similar APOE4 carrier rate was found between the two groups (345 [51.7%] for OP−; 26 [52.0%] for OP+; *P* = 1.000). Education years were approximately 6 months shorter for participants with OP (16.0 [14.0, 18.0] for OP−; 15.5 [13.0, 18.0] for OP+; *P* = 0.108). In short, only “sex” was found to be significantly different between the two groups.

We further compared the CSF Aβ_1–42_, t-tau, and p-tau_181_ between the two groups at baseline. The OP− group had a lower Aβ_1–42_ (763.1 [566.4, 1112.8], pg/ml) than the OP + group (792.2 [619.5, 989.8], pg/ml; *P* = 0.841). Both t-tau and p-tau_181_ were significantly lower in OP− group than OP + group (253.6 [186.6, 347.6] vs. 324.8 [237.2, 438.2] for t-tau, pg/ml; *P* = 0.003; 24.4 [16.6, 35.4] vs. 31.8 [21.8, 44.1] for p-tau_181_, pg/ml; *P* = 0.005).

#### Brain structures

In the MRI data, at baseline, the measurements of ECV, HV, and ICV were available for 992 participants, of whom 81 had OP (274 [30.1%] NC, 492 [54.0%] MCI, and 145 [15.9%] AD in OP− group; 20 [24.7%] NC, 44 [54.3%] MCI, and 17 [21.0%] AD in OP + group; *P* = 0.387). Participants with OP were roughly 2 years older than those without OP (73.7 [6.9] for OP−, 75.7 [7.0] for OP+; *P* = 0.013). Sex composition differed significantly between the two groups (female, OP−: 337 [37.0%] vs. OP+: 69 [85.2%]; *P* < 0.001). There was no significant difference in the APOE4 carrier rate between the two groups (423 [46.4%] for OP−; 36 [44.4%] for OP+; *P* = 0.820). Education years between the two groups were very close (OP−: 16.0 [14.0, 18.0] vs. OP+: 16.0 [14.0, 18.0]; *P* = 0.651). To sum up, “age” and “sex” were found to be significantly different between the two groups.

We further examined the ECV_*n*_ and HV_*n*_ between the two groups at baseline. Both ECV_*n*_ and HV_*n*_ were higher in the OP− group than in the OP + group (3,537.1 [756.2] vs. 3,375.8 [746.9] for ECV_*n*_, mm^3^; *P* = 0.066; 6,898.1 [1,145.8] vs. 6,558.3 [949.9] for HV_*n*_, mm^3^; *P* = 0.010). However, the difference was only significant for HV_*n*_.

Table 1 provides an overview of the baseline CSF and MRI data, in which the differences between the two groups are summarized.

**TABLE 1:** Participant characteristics at baseline.

		CSF data			MRI data		
	Level	OP−	OP+	*P*-value	OP−	OP+	*P*-value
Number of participants (n)		667	50		911	81	
Age (mean [SD]) (years)		73.9 [7.2]	74.9 [7.0]	0.382^t^	73.7 [6.9]	75.7 [7.0]	**0.013** ^t^
Sex [%]	Female	233 [34.9]	42 [84.0]	**<0.001** ^c^	337[37.0]	69 [85.2]	**<0.001** ^c^
	Male	434 [65.1]	8 [16.0]		574 [63.0]	12 [14.8]	
Education (median [P25, P75]) (years)		16.0 [14.0, 18.0]	15.5 [13.0, 18.0]	0.108^w^	16.0 [14.0, 18.0]	16.0 [14.0, 18.0]	0.651^w^
Diagnosis (n [%])	NC	154 [23.1]	8 [16.0]	0.256^c^	274 [30.1]	20 [24.7]	0.387^c^
	MCI	383 [57.4]	28 [56.0]		492 [54.0]	44 [54.3]	
	AD	130 [19.5]	14 [28.0]		145 [15.9]	17 [21.0]	
APOE4 (n [%])	APOE4-	322 [48.3]	24 [48.0]	1.000^c^	488 [53.6]	45 [55.6]	0.820^c^
	APOE4 +	345 [51.7]	26 [52.0]		423 [46.4]	36 [44.4]	
ECV_n_ (mean [SD]) (mm^3^)					3,537.1 [756.2]	3,375.8 [746.9]	0.066^t^
HV_n_ (mean [SD]) (mm^3^)					6,898.1 [1,145.8]	6,558.3 [949.9]	**0.010** ^t^
Aβ_1–42_ (median [P25, P75]) (pg/ml)		763.1 [566.4, 1,112.8]	792.2 [619.5, 989.8]	0.841^w^			
t-tau (median [P25, P75]) (pg/ml)		253.6 [186.6, 347.6]	324.8 [237.2, 438.2]	**0.003** ^w^			
p-tau_181_ (median [P25, P75]) (pg/ml)		24.4 [16.6, 35.4]	31.8 [21.8, 44.1]	**0.005** ^w^			

*Baseline demographic variables were compared between the OP− and OP+ groups. ^c^, Chi-square tests for categorical variables; ^t^, Student’s t-tests for normally distributed variables; ^w^, Mann–Whitney U-tests for non-normally distributed variables. OP, osteoporosis; NC, normal control; MCI, mild cognitive impairment; AD, Alzheimer’s disease; APOE4, apolipoprotein E4; ECV_n_, intracranial volume normalized entorhinal cortex volume; HV_n_, intracranial volume normalized hippocampal volume; Aβ_1–42_, amyloid-beta 1-42; t-tau, total tau; p-tau_181_, tau phosphorylated at threonine-181. Bold font indicates statistical significance.*

### Cross-sectional analyses at baseline

**FIGURE 2:**
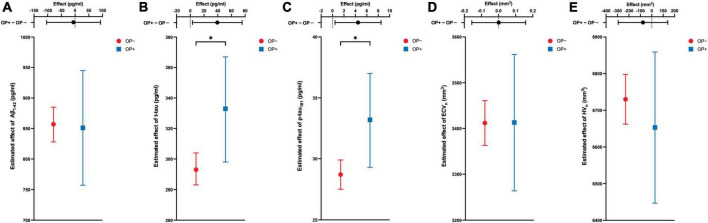
The cross-sectional associations between OP and the response variable of the model. Estimated average effects with 95% CI error bars are demonstrated on each panel. The upper part of each panel represents the contrast (mean of OP + group minus mean of OP– group) with a 95% CI error bar. OP is associated with cerebrospinal fluid levels of t-tau and p-tau_181_ **(B,C)**, but not associated with the Aβ_1–42_ **(A)**, ECV_n_ **(D)**, and HV_n_ **(E)**. All analyses are adjusted for age, gender, education, APOE4 carrier status, and diagnosis of Alzheimer’s disease (AD). *P-value <0.05. OP, osteoporosis; Aβ_1–42_, amyloid-beta 1-42; t-tau, total tau; p-tau_181_, tau phosphorylated at threonine-181; ECV_n_, intracranial volume normalized entorhinal cortex volume; HV_n_, intracranial volume normalized hippocampal volume.

To evaluate the relationship between OP and AD at baseline, CSF biomarkers and brain structural volumes were adjusted for five covariates, including age, sex, education, APOE4 carrier status, and AD diagnosis *via* linear regression models. Briefly speaking, OP was significantly linked to a higher estimated average effect of CSF t-tau and p-tau_181_ at baseline (estimate = 39.4 pg/ml, 95% CI: 3.2–75.5 pg/ml, *P* = 0.033 for t-tau; estimate = 4.5 pg/ml, 95% CI: 0.4–8.5 pg/ml, *P* = 0.030 for p-tau_181_). ECV_*n*_ was almost irrelevant with OP (estimate = 0.7 mm^3^, 95% CI: −156.2–157.6 mm^3^, *P* = 0.993; mm^3^). Baseline estimated average effect of CSF Aβ_1–42_ and HV_*n*_ were slightly lower in OP + group, but not statistically significant (estimate = –5.9 pg/ml, 95% CI: −104.4–92.7 pg/ml, *P* = 0.907 for Aβ_1–42_; estimate = −77.1 mm^3^, 95% CI: −293.8–139.6 mm^3^, *P* = 0.485 for HV_*n*_). The simplified outcomes of these models are presented in Table 2 and are visualized in Figure 2. The detailed results (including covariates) are available in Supplementary Table 1.

**TABLE 2:** Summary of linear regression models examining the association of OP status with the response variables.

Response variable	Independent variable	Estimate	95% CI	*P*-value
Aβ_1–42_ (pg/mL)	OP status (OP+)	−5.9	−104.4 to 92.7	0.907
t-tau (pg/mL)	OP status (OP+)	39.4	3.2–75.5	**0.033**
p-tau_181_ (pg/mL)	OP status (OP+)	4.5	0.4–8.5	**0.030**
ECV_n_ (mm^3^)	OP status (OP+)	0.7	−156.2 to 157.6	0.993
HV_n_ (mm^3^)	OP status (OP+)	−77.1	−293.8 to 139.6	0.485

*OP status (OP+) is significantly associated with t-tau and p-tau_181_ at baseline. OP, osteoporosis; Aβ_1–42_, amyloid-beta 1-42; t-tau, total tau; p-tau_181_, tau phosphorylated at threonine-181; ECV_n_, intracranial volume normalized entorhinal cortex volume; HV_n_, intracranial volume normalized hippocampal volume. Bold font indicates statistical significance.*

### Longitudinal analyses

**FIGURE 3:**
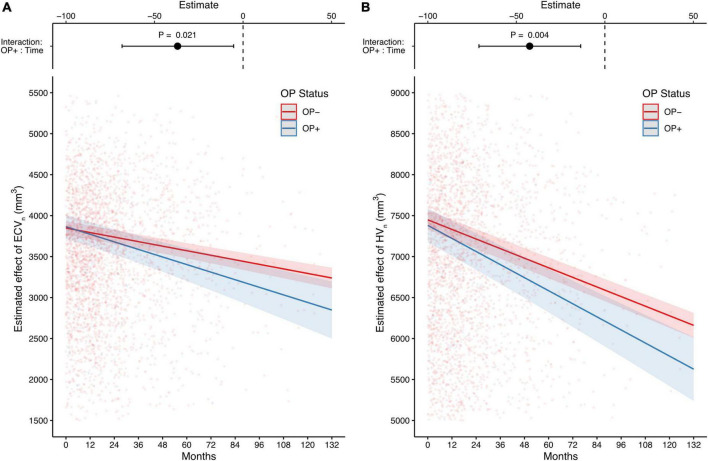
The long-term effects of OP on entorhinal cortex and hippocampus MRI-based measurements. Compared with individuals without OP, those with OP show a faster decline in both the estimated effect of ECV_n_ and HV_n_ **(A,B)**. The upper part of each panel represents the contrast of the predictor (the interaction of OP+ and time; mean of OP + group minus mean of OP– group) with a 95% CI error bar. Both analyses are adjusted for age, gender, education, APOE4 carrier status, and diagnosis of Alzheimer’s disease (AD). OP, osteoporosis; ECV_n_, intracranial volume normalized entorhinal cortex volume; HV_n_, intracranial volume normalized hippocampal volume.

After completing the cross-sectional analyses, we investigated how OP affected the changes in CSF biomarkers and brain structural volumes over time. Unlike the cross-sectional results, CSF biomarkers were not associated with OP status over time (Table 3). By contrast, compared with individuals without OP, those with OP showed significantly faster declines in both ECV_*n*_ and HV_*n*_ (estimate = −37 mm^3^, 95% CI: –68.3 to –5.6 mm^3^, *P* = 0.021 for ECV_*n*_ on interaction of OP and time [OP+: time], Figure 3A; estimate = −42.5 mm^3^, 95% CI: −71.1 to –13.8 mm^3^, *P* = 0.004 for HV_n_ on interaction of OP and time [OP+: time], Figure 3B). Detailed longitudinal results (including covariates) are provided in Supplementary Table 2.

**TABLE 3:** Summary of linear mixed-effects models examining the association of OP status with changes in response variables over time.

Response variable of model	Predictor	Estimate	95% CI	*P*-value
Aβ_1–42_ (pg/mL)	Time: OP+	4.5	−15.0 to 24.1	0.648
	Time	−13.5	−19.4 to −7.6	**<0.001**
	OP status (OP+)	12.1	−114.2 to 138.4	0.851
t-tau (pg/mL)	Time: OP+	4.2	−1.8 to 10.3	0.17
	Time	5.1	3.4 to 6.8	**<0.001**
	OP status (OP+)	11.4	−37.3 to 60.2	0.645
p-tau_181_ (pg/mL)	Time: OP+	0	−0.7 to 0.6	0.902
	Time	0.4	0.2 to 0.6	**<0.001**
	OP status (OP+)	1.3	−4.2 to 6.8	0.633
ECV_n_ (mm^3^)	Time: OP+	−37	−68.3 to −5.6	**0.021**
	Time	−55.5	−64.4 to −46.5	**<0.001**
	OP status (OP+)	15.8	−138.1 to 169.7	0.841
HV_n_ (mm^3^)	Time: OP+	−42.5	−71.1 to −13.8	**0.004**
	Time	−117.1	−125.1 to −109.1	**<0.001**
	OP status (OP+)	−67.5	−283.7 to 148.7	0.54

*The interaction of time and OP status (Time: OP+) was the main predictor of the linear mixed-effects model. The interaction represents the relationship between OP status (OP+) and the change of response variable over time. The changes of ECV_n_ and HV_n_ over time are both significantly associated with OP. OP, osteoporosis; Aβ_1–42_, amyloid-beta 1-42; t-tau, total tau; p-tau_181_, tau phosphorylated at threonine-181; ECV_n_, intracranial volume normalized entorhinal cortex volume; HV_n_, intracranial volume normalized hippocampal volume. Bold font indicates statistical significance.*

In addition, as “loss to follow-up” aggravated with time in both groups, a series of sensitivity tests were conducted on the data with follow-up cutoffs from 24 to 132 (maximum) months. We found no decisive differences in outcomes when the follow-up cutoff time exceeded 48 months. For each time point, the numbers of follow-up visits are listed, along with the *P*-values of sensitivity tests (Supplementary Table 3, full results not shown).

## Discussion

Alzheimer’s Disease Neuroimaging Initiative is a historic study of brain aging that aims to accelerate discovery in the race to prevent, treat, and eventually cure AD. For more than a decade, ADNI researchers have been working to better understand AD. In addition to studying AD itself, many researchers, including ourselves, are interested in investigating the links between AD and other diseases, such as osteoarthritis (Li et al., 2020), Parkinson’s disease (Wang et al., 2018), hearing loss (Xu et al., 2019), hypertension (Scott et al., 2015; Zhou et al., 2020), and hypercholesterolemia (Varma et al., 2021), from the ADNI database. To the best of our knowledge, this is the first study to demonstrate the links between OP and higher levels of CSF t-tau and p-tau_181_ at baseline, and the faster volumetric declines of enterhinal cortex and hippocampus over time among older people.

Our findings are consistent with previous studies on tau pathology. Histopathological data showed a significantly reduced BMD phenotype was found in the htau mouse models (Dengler-Crish et al., 2017). Dengler-Crish et al. (2018) further reported that the BMD reduction occurred before the presence of significant tauopathy in the hippocampus, which can be implied that OP status may risk neurodegeneration *via* promoting tau pathology. Tauopathy could induce cognitive impairment across the AD spectrum *via* synaptic dysfunction and neuronal loss (Andorfer et al., 2003; Polydoro et al., 2009; Di et al., 2016), independent of amyloid pathology (Bejanin et al., 2017). Notably, the weak links we found between OP and the buildup of CSF t-tau and p-tau_181_ over time were inconsistent with Dengler-Crish’s study (Dengler-Crish et al., 2018). These conflicts may be partially explained by sample removal for longitudinal CSF data analyses, which is reasonable given that the CSF collection is invasive and the subjects are real humans. Still, it is hoped that future research will provide more comprehensive longitudinal data to validate our results.

Interestingly, subjects with OP began with comparable baseline volumes as those without OP and experienced a significantly faster decline in both ECV_n_ and HV_n_ over time. In addition, sensitivity tests’ outcomes barely changed when they were tracked for more than 48 months. The possible explanations are as follows: (1) OP might play different roles in different stages of the AD continuum; (2) the individual variability of ECV_n_ and HV_n_ at baseline was low; and (3) the process of brain structural atrophy caused by abnormal accumulation of CSF pathological proteins takes a long time (Killiany et al., 2002; Kril et al., 2002; Dengler-Crish et al., 2018). These explanations merit further studies.

There are several possible molecular biological explanations for our findings. Currently known, pathways that OP and AD may share include RANK-RANKL (Luckhaus et al., 2009; Stapledon et al., 2021), vitamin D receptor (Morello et al., 2018; Jia et al., 2019), C/EBPβ-δ-secretase (Xiong et al., 2022), PI3K-Akt (Fehsel and Christl, 2022), and Wnt/β-catenin (Li et al., 2013; Dengler-Crish et al., 2018; Folke et al., 2019), among which Wnt/β-catenin had the most attention. Wnt/β-catenin signaling is known to facilitate bone formation in bone tissue and to promote synaptogenesis in the brain. Likewise, pathological inhibition of this pathway has been implicated in both OP and AD pathogenesis, albeit in separate contexts (Dengler-Crish and Elefteriou, 2019). More importantly, Wnt deficiency was detectable in bone prior to the brain in the htau mouse model (Dengler-Crish et al., 2018), which may help explain our findings and emphasize the necessity of OP screening in AD-susceptible populations. Aside from Wnt/β-catenin signaling, RANK-RANKL signaling is another pathway shared by both OP and AD. According to Li et al. (2016) Aβ enhances RANKL-induced osteoclast activation and function, implying that Aβ is involved in the pathogenesis of OP at molecular levels in osteoclasts. Furthermore, the link between vitamin D deficiency and AD is gaining attention. Vitamin D supplementation improved cognitive function in both the AD mouse model (Morello et al., 2018) and in a randomized, double-blind, and placebo-controlled trial in which Aβ-related biomarkers were found to be lower in elderly patients with AD (Jia et al., 2019). Our findings, however, did not support these Aβ-related pathophysiological explanations. These discrepancies may be partially explained by differences in Aβ distribution and accumulation between the central nervous system and peripheral tissues (Li et al., 2014; Roberts et al., 2014; Ristori et al., 2020). More studies are needed to elucidate the potential mechanisms through which OP acts in AD.

Our study has certain limitations. First, the attrition bias due to loss to follow-up is not corrected in the analyses. Furthermore, the sample size is not balanced between the two groups. Future studies with larger sample size, longer follow-up duration, and lower attrition rates might provide more powerful evidence to support our findings. Second, the ADNI database does not contain adequate information about OP, such as the time OP started, lab evidence, or imaging evidence, for every participant with OP or without OP. This could lead to some wrong classifications. Even so, this is a common limitation of database-based research (Norton et al., 2014; Frain et al., 2017; Xu et al., 2019; Harshfield et al., 2020; Li et al., 2020; Kwon et al., 2021). Future studies are encouraged to extend our work to other databases to verify our results. Third, the associations we discovered can reflect but not represent causal relationships. Direct evidence of causal relationships may be provided by future research focusing on the protective effect of anti-osteoporosis drugs, such as bisphosphonates, on AD development. Fourth, although we excluded individuals with psychiatric conditions other than AD in the present analyses, we still cannot exclude the influence of other potential confounders, such as social isolation, eating disorder, or addictive behavior. Moreover, our models included five covariates. Other potential covariates (e.g., hypertension and hyperlipemia) are not included since they are recorded either binomially (without specific values) or merely once. Adding them to our models may not improve accuracy but will increase complexity and inefficiency (Zhang, 2014). Future research can be devoted to adding more valuable covariates, but this will need better database support.

## Conclusion

This study identified the cross-sectional and longitudinal association between OP and the known pathological features of AD. Our findings suggest that OP’s neurodegenerative effects may be driven by elevated baseline CSF t-tau and p-tau levels, and the accelerated entorhinal cortex and hippocampal atrophy among older adults, providing critical insight into the neuropathological mechanisms by which OP increases the risk of developing AD. Furthermore, our results suggest that preventing or managing OP during the preclinical and prodromal stages of AD may be effective in combating neurodegeneration among older people.

## Data availability statement

The original contributions presented in this study are included in the article/Supplementary material, further inquiries can be directed to the corresponding author/s.

## Ethics statement

The studies involving human participants were reviewed and approved by Alzheimer’s Disease Neuroimaging Initiative. The patients/participants provided their written informed consent to participate in this study.

## Author contributions

YL designed and supervised the study. HP, JC, CW, PW, JL, and FH performed the research, analyzed the data, and wrote the manuscript. All authors contributed to the article and approved the submitted version.

## Footnotes

1
adni.loni.usc.edu
