# Supplementary material for: Osteoporosis is associated with elevated baseline cerebrospinal fluid biomarkers and accelerated brain structural atrophy among older people
Source: Front Aging Neurosci. 2022 Sep 16;14:958050. doi: 10.3389/fnagi.2022.958050 (PMC9523506; doi:10.3389/fnagi.2022.958050)
Supplement: Supplementary file 1 [file Data_Sheet_1.docx]

**Supplementary Table 1** | The summary of cross-sectional regression results between OP and response variables with covariates at baseline.

|  | | **Aβ_1-42_ (pg/mL)** | | | **t-tau (pg/mL)** | | | **p-tau_181_ (pg/mL)** | | | **ECV_n_ (mm^3^)** | | | **HVn (mm^3^)** | | |
| --- | --- | --- | --- | --- | --- | --- | --- | --- | --- | --- | --- | --- | --- | --- | --- | --- |
|  | | **Estimate** | **95% CI** | **P value** | **Estimate** | **95% CI** | **P value** | **Estimate** | **95% CI** | **P value** | **Estimate** | **95% CI** | **P value** | **Estimate** | **95% CI** | **P value** |
| Independent variable | OP status (OP+) | -5.9 | -104.4 ~ 92.7 | 0.907 | 39.4 | 3.2 ~ 75.5 | **0.033** | 4.5 | 0.4 ~ 8.5 | **0.030** | 0.7 | -156.2 ~ 157.6 | 0.993 | -77.1 | -293.8 ~ 139.6 | 0.485 |
| Covariates | Age | -3.4 | -6.8 ~ 0.1 | 0.054 | 1.9 | 0.7 ~ 3.2 | **0.003** | 0.2 | 0.0 ~ 0.3 | **0.020** | -21.0 | -27.1 ~ -15.0 | **<0.001** | -61.0 | -69.4 ~ -52.6 | **<0.001** |
|  | Education | -0.6 | -9.9 ~ 8.6 | 0.893 | -2.0 | -5.4 ~ 1.4 | 0.254 | -0.2 | -0.6 ~ 0.1 | 0.223 | 14.5 | -0.6 ~ 29.6 | 0.059 | 12.7 | -8.1 ~ 33.6 | 0.231 |
|  | Sex (Male) | -26.1 | -79.7 ~ 27.6 | 0.341 | -31.6 | -51.3 ~ -11.9 | **0.002** | -3.2 | -5.4 ~ -1.0 | **0.005** | 148.3 | 58.8 ~ 237.7 | **0.001** | 142.7 | 19.1 ~ 266.2 | **0.024** |
|  | APOE4 (+) | -243.3 | -293.7 ~ -192.8 | **<0.001** | 56.7 | 38.2 ~ 75.2 | **<0.001** | 6.5 | 4.4 ~ 8.5 | **<0.001** | -177.7 | -264.5 ~ -91.0 | **<0.001** | -320.0 | -439.9 ~ -200.2 | **<0.001** |
|  | Diagnosis | -193.7 | -248.4 ~ -139.1 | **<0.001** | 85.1 | 65.1 ~ 105.1 | **<0.001** | 9.4 | 7.2 ~ 11.7 | **<0.001** | -646.9 | -741.4 ~ -552.4 | **<0.001** | -1012.4 | -1142.9 ~ -881.9 | **<0.001** |
| R^2^ adjusted | | 0.20 | | | 0.19 | | | 0.19 | | | 0.24 | | | 0.36 | | |

OP status (OP+) is significantly associated with t-tau and p-tau_181_ at baseline. Abbreviations: OP = osteoporosis; Aβ_1-42_ = amyloid-beta_1-42_; t-tau = total tau; p-tau_181_= tau phosphorylated at threonine-181; ECV_n_ = intracranial volume normalized entorhinal cortex volume; HV_n_ = intracranial volume normalized hippocampal volume; APOE4 = apolipoprotein E4.

**Supplementary Table 2** | The summary of longitudinal regression results between OP and the predictors with covariates.

|  | | **Aβ_1-42_ (pg/mL)** | | | **t-tau (pg/mL)** | | | **p-tau_181_ (pg/mL)** | | | **ECV_n_ (mm^3^)** | | | **HV_n_ (mm^3^)** | | |
| --- | --- | --- | --- | --- | --- | --- | --- | --- | --- | --- | --- | --- | --- | --- | --- | --- |
|  | | **Estimate** | **95% CI** | **P-value** | **Estimate** | **95% CI** | **P value** | **Estimate** | **95% CI** | **P-value** | **Estimate** | **95% CI** | **P-value** | **Estimate** | **95% CI** | **P-value** |
| Predictor | Time : OP (OP+) | 4.5 | -15.0 ~ 24.1 | 0.648 | 4.2 | -1.8 ~ 10.3 | 0.17 | 0 | -0.7 ~ 0.6 | 0.902 | -37 | -68.3 ~ -5.6 | **0.021** | -42.5 | -71.1 ~ -13.8 | **0.004** |
|  | OP status (OP+) | 12.1 | -114.2 ~ 138.4 | 0.851 | 11.4 | -37.3 ~ 60.2 | 0.645 | 1.3 | -4.2 ~ 6.8 | 0.633 | 15.8 | -138.1 ~ 169.7 | 0.841 | -67.5 | -283.7 ~ 148.7 | 0.54 |
|  | Time | -13.5 | -19.4 ~ -7.6 | **<0.001** | 5.1 | 3.4 ~ 6.8 | **<0.001** | 0.4 | 0.2 ~ 0.6 | **<0.001** | -55.5 | -64.4 ~ -46.5 | **<0.001** | -117.1 | -125.1 ~ -109.1 | **<0.001** |
| Covariates | Age | -1.3 | -5.8 ~ 3.3 | 0.586 | 2.2 | 0.4 ~ 3.9 | **0.017** | 0.2 | -0.0 ~ 0.4 | 0.079 | -23 | -28.9 ~ -17.0 | **<0.001** | -60 | -68.4 ~ -51.7 | **<0.001** |
|  | Education | 3.4 | -8.0 ~ 14.8 | 0.556 | -0.5 | -5.0 ~ 3.9 | 0.811 | 0 | -0.5 ~ 0.5 | 0.938 | 16.2 | 1.6 ~ 30.9 | **0.03** | 12.3 | -8.3 ~ 32.9 | 0.243 |
|  | Sex (Male) | -281.1 | -344.0 ~ -218.2 | **<0.001** | 55.8 | 31.3 ~ 80.3 | **<0.001** | 6.3 | 3.6 ~ 9.1 | **<0.001** | -201.5 | -285.9 ~ -117.1 | **<0.001** | -284.5 | -403.0 ~ -165.9 | **<0.001** |
|  | APOE4 (+) | -13 | -81.9 ~ 55.9 | 0.711 | -32.4 | -59.3 ~ -5.6 | **0.018** | -3.9 | -6.9 ~ -0.8 | **0.013** | 148.5 | 61.5 ~ 235.5 | **0.001** | 133.9 | 11.7 ~ 256.1 | **0.032** |
|  | Diagnosis | -196.1 | -269.4 ~ -122.8 | **<0.001** | 80.6 | 52.0 ~ 109.2 | **<0.001** | 9.4 | 6.2 ~ 12.6 | **<0.001** | -657.5 | -749.8 ~ -565.3 | **<0.001** | -997.8 | -1127.5 ~ -868.0 | **<0.001** |
| Adjusted R**^2^** |  | 0.91 | | | 0.97 | | | 0.97 | | | 0.87 | | | 0.98 | | |

The five linear mixed effects models characterize individual paths of change (models for Aβ_1-42_, t-tau, p-tau_181_, ECV_n_, and HV_n_). They had random intercepts and slopes for time and an unstructured covariance matrix for the random effects. The interaction of time and OP status (Time : OP+) was the main predictor of these models. It represents the relationship between OP status (OP+) and the change of response variable over time. These models were adjusted for the same covariates, including age, sex, education, APOE4 carrier status, and diagnosis of AD. Abbreviations: OP = osteoporosis; Aβ_1-42_ = amyloid-beta_1-42_; t-tau = total tau; p-tau_181_= tau phosphorylated at threonine-181; APOE4 = apolipoprotein E4; ECV_n_ = intracranial volume normalized entorhinal cortex volume; HV_n_ = intracranial volume normalized hippocampal volume.

**Supplementary Table 3** | Follow-up visit and the result of sensitivity test.

| **Time (Months)** | **Follow-up visits (n subjects)** | | | | **P-value of the sensitivity test** | | | | |
| --- | --- | --- | --- | --- | --- | --- | --- | --- | --- |
|  | **CSF data** | | **MRI data** | | **Aβ1-42** | **t-tau** | **p-tau181** | **ECV_n_** | **HV_n_** |
|  | **OP−** | **OP+** | **OP−** | **OP+** |  |  |  |  |  |
| 0 | 347 | 25 | 911 | 81 | SF | | | | |
| 6 | 0 | 0 | 662 | 49 |  | | | | |
| 12 | 172 | 21 | 747 | 63 |  | | | | |
| 24 | 207 | 11 | 507 | 44 | SF | SF | 0.854 | SF | SF |
| 36 | 42 | 7 | 209 | 23 | SF | SF | 0.355 | 0.658 | 0.200 |
| 48 | 87 | 5 | 220 | 11 | 0.982 | 0.932 | 0.325 | 0.369 | 0.117 |
| 60 | 19 | 2 | 90 | 9 | 0.558 | 0.312 | 0.819 | **0.036** | **0.026** |
| 72 | 11 | 4 | 85 | 8 | 0.763 | 0.072 | 0.813 | **0.012** | **0.006** |
| 84 | 7 | 1 | 17 | 3 | 0.544 | 0.054 | 0.664 | **0.025** | **0.004** |
| 96 | 3 | 1 | 17 | 1 | 0.574 | 0.093 | 0.883 | **0.020** | **0.003** |
| 108 | 7 | 1 | 14 | 1 | 0.582 | 0.093 | 0.882 | **0.023** | **0.003** |
| 120 | 2 | 0 | 8 | 1 | 0.656 | 0.173 | 0.901 | **0.021** | **0.003** |
| 132 | 0 | 0 | 1 | 0 | 0.650 | 0.171 | 0.902 | **0.021** | **0.004** |

Follow-up visit numbers of subjects and the P-values of the main predictor (interaction of OP+ and time) in sensitivity tests are listed at each visit/cutoff time. When the follow-up cutoff time exceeded 48 months, P-value in the columns of ECV_n_ and HV_n_ are all smaller than 0.05. Abbreviations: SF = Singular fit (SF means the model is overfitted, that is, the random effects structure is too complex to be supported by the data.); OP = osteoporosis; Aβ_1-42_ = amyloid-beta_1-42_; t-tau = total tau; p-tau_181_ = tau phosphorylated at threonine-181; ECV_n_ = intracranial volume normalized entorhinal cortex volume; HV_n_ = intracranial volume normalized hippocampal volume.
